# Hepatitis C virus testing in a clinical HIV cohort in Ontario, Canada, 2000 to 2015

**DOI:** 10.1002/hsr2.358

**Published:** 2021-09-18

**Authors:** Nasheed Moqueet, Ramandip Grewal, Tony Mazzulli, Curtis Cooper, Sandra L. Gardner, Irving E. Salit, Abigail Kroch, Ann N. Burchell, OHTN Cohort Study Team, Abigail Kroch, Ann Burchell, Sergio Rueda, Gordon Arbess, Jeffrey Cohen, Curtis Cooper, Elizabeth Lavoie, Fred Crouzat, Nisha Andany, Sharon Walmsley, Michael Silverman, Roger Sandre, Wangari Tharao, Holly Gauvin, Fiona Smaill, Jorge Martinez‐Cajas

**Affiliations:** ^1^ MAP Centre for Urban Health Solutions, Li Ka Shing Knowledge Institute, St. Michael's Hospital Unity Health Toronto Toronto Ontario Canada; ^2^ Department of Microbiology Mount Sinai Hospital and University Health Network Toronto Ontario Canada; ^3^ Public Health Ontario Toronto Ontario Canada; ^4^ Toronto General Hospital University Health Network Toronto Ontario Canada; ^5^ Department of Laboratory Medicine and Pathobiology University of Toronto Toronto Ontario Canada; ^6^ The Ottawa Hospital‐Division of Infectious Diseases Ottawa Ontario Canada; ^7^ Dalla Lana School of Public Health University of Toronto Toronto Ontario Canada; ^8^ Rotman Research Institute Toronto Ontario Canada; ^9^ The Ontario HIV Treatment Network Toronto Ontario Canada; ^10^ Department of Family and Community Medicine, Faculty of Medicine University of Toronto Toronto Ontario Canada

**Keywords:** Coinfection/epidemiology, hepatitis C/epidemiology*, hepatitis C virus testing, HIV infections/epidemiology*, HIV‐HCV co‐infection

## Abstract

**Background:**

HIV‐positive individuals may acquire HCV via injection drug use (IDU) and condomless anal sex. HIV care provides opportunities for HCV testing and cure with direct‐acting antiviral agents (DAAs).

**Methods:**

We analyzed data from the Ontario HIV Treatment Network Cohort Study. Among those not HCV‐positive or diagnosed previously (n = 4586), we used Cox regression to test the rates of ever HCV testing (serological or RNA) in HIV care by DAA era (pre‐DAA: 2000‐2010; after DAA: 2011‐2015) and compared the proportion diagnosed with HCV. We identified correlates of annual proportions of serological testing using Poisson generalized estimating equations.

**Results:**

After DAA vs pre‐DAA, the hazard rate ratio (95% CI) of ever HCV testing was 1.70 (1.59, 1.81). The proportion (95% CI) tested annually increased from 9.2% (8.0%, 10.7%) in 2000 to 39.1% (37.1%, 41.1%) in 2015 (*P* < 0.0001). The proportion diagnosed with HCV declined by 74% pre‐DAA to 11% after DAAs. Annual testing increased per calendar year (16% steeper slope after DAA vs pre‐DAA) and was more common among men who have sex with men; those more educated (post‐secondary vs ≤ high school); and those positive for syphilis or reporting any IDU. Annual testing decreased per decade of age and time since HIV diagnosis.

**Discussion:**

Annual HCV testing increased over time with higher testing among those reporting sexual or IDU risk factors, but fell short of clinical guidelines. Targeted interventions to boost testing may be needed to close these gaps and reach WHO 2030 HCV elimination targets.

## 1 INTRODUCTION

People living with HIV are vulnerable to the acquisition and consequences of Hepatitis C virus (HCV) co‐infection due to biological and social factors. In Canada, 18% to 20% of those living with HIV are co‐infected with HCV compared to <1% in the general population.^1^, ^2^ Direct‐acting antiviral (DAA) drugs, approved by Health Canada in 2011, are highly efficacious and curative (>95%) even in co‐infected individuals, though uptake was initially low. Timely HCV testing and treatment with DAA drugs can ameliorate clinical complications and interrupt ongoing HCV transmission, thus making it possible to reach elimination goals set by the World Health Organization (WHO) by 2030 (90% diagnosis, 80% reduction in HCV incidence, and 65% reduction in HCV‐related mortality).^3^

In 1999, U.S. guidelines first recommended testing all HIV‐positive individuals for HCV ^4^ though HCV testing in Canada remained risk or symptom based even after approval of DAAs in 2011 and interferon‐free, all‐oral regiments in 2014.^5^ It was not until 2016 that new Canadian guidelines recommended one‐time HCV testing when first evaluated for HIV, followed by annual retesting for “high risk” individuals such as people actively injecting drugs and sexually active HIV‐positive men who have sex with men (MSM) engaging in “high risk” behaviors.^6^

In 2011, 44% of those chronically infected with HCV were estimated to be undiagnosed in Canada.^7^ Diagnosis rates were thought to be higher for people living with HIV, as attending HIV care provides opportunities for HCV screening and treatment. To our knowledge, there are no published reports of HCV testing in this population in Canada, which could provide historical estimates for inputs of mathematical models of HCV transmission and identify gaps in HCV care cascades and barriers to reaching WHO elimination targets. Therefore, we sought to characterize temporal patterns of HCV testing between 2000 and 2015 in a clinical cohort of HIV patients in Ontario, the province that comprised 38.7% of all reported HCV cases in Canada in 2009 and has the largest population of people living with HIV.^2^ We aimed to estimate the annual proportion that had tested for HCV, the frequency of annual serological HCV tests, and the proportion diagnosed with HCV. We hypothesized that HCV testing trends would reflect the testing guidelines and treatment options over time, with higher testing after DAA approval (2011 onwards) and in groups perceived to be at higher risk for HCV acquisition.

## 2 METHODS

We used data from the open, prospective Ontario HIV Treatment Network Cohort Study (OCS), which has been described previously.^8^ Briefly, the OCS represents almost 25% of the HIV patients under care in Ontario, consisting of participants aged 16 or older who volunteered to be a part of the study and accessed HIV care at any of nine participating clinics. From 1995 to 2007, participants self‐completed a questionnaire at enrollment; since 2008, they were interviewed annually.^8^ Clinical data was abstracted from medical charts. The study protocol, research instruments and forms received ethical approval from the University of Toronto Human Subjects Review Committee and from the individual study sites.

### 2.1 Testing and laboratory data

We obtained testing data for HIV viral load, HCV and syphilis through linkage with OCS clinical records and the provincial Public Health Ontario Laboratories (PHOL), the sole provider of HIV viral load and syphilis serological tests in Ontario. In Ontario, HCV serological testing can be performed by private laboratories, hospital laboratories, or at the PHOL. However, almost all confirmatory HCV serological tests and HCV‐RNA detection and quantification are conducted by the PHOL. Testing for HCV antibodies (anti‐HCV) is often the first recommended step in the testing algorithm, and if positive, is followed by HCV RNA detection, measurement and genotyping. We defined “ever testers” as those with at least one HCV test (serological or RNA), and “annual testers” as those with at least 1 HCV serological test in a calendar year that they were under observation (Details in Table S1). HCV diagnosis was classified on the basis of laboratory test results (confirmed antibody test or positive RNA or genotype test) or notation of an HCV diagnosis in a participant's medical record.

### 2.2 Inclusion criteria for analysis

As of December 2015, a total of 6891 participants had enrolled in the OCS. We restricted the analyses to participants who had at least linked 1 HIV viral load test and were enrolled in the OCS between 2000 and 2015 (1731 removed) so that any HCV testing with the PHOL would be captured. Accumulation of study time began at ***baseline***, which we defined as the later of January 1, 2000, the date of first HIV viral load test or the date of the first OCS visit. Because we were interested in HCV testing patterns among people in HIV care who were not yet known to have HCV, we excluded those who had been diagnosed or tested positive for HCV prior to baseline (574 removed) based on either HCV diagnosis dates on medical records or positive HCV antibody, RNA, or genotype tests (remaining analytic sample, n = 4586). Additional restrictions for specific analyses are described below and summarized in Table S1. All statistical analyses were conducted with Stata v13 (College Station, TX) ^9^ and we used a complete case analysis strategy to handle missing data.

### 2.3 Covariates

Temporal trends were assessed per calendar year with a linear spline knot at 2011, when DAAs were approved by Health Canada (ie, pre‐DAA era = 2000‐2010 vs DAA era = 2011‐2015). Any history of injection drug use (IDU), included as a dichotomous measure, was based on participants' HIV exposure category or any self‐reported IDU in annual questionnaires, which inquired about any non‐medicinal IDU prior to HIV diagnosis or in the past 6 months. For a subanalysis, recent IDU, referring to IDU in the past 6 months, was available as a binary time‐varying variable only among a subset of participants who completed annual questionnaires after 2008.

Self‐reported binary variables were used for sex and MSM. Ethnicity was categorized as white, black, Aboriginal/indigenous, other, or unknown. Based on previous analyses of HCV seroconversion among MSM in the OCS,^10^ we considered having ever had syphilis as a proxy measure of high‐risk sexual behavior for HCV acquisition; this was defined as a dichotomous record of any reactive syphilis test result. We analyzed age and HIV duration by decade, where the latter was based on an estimated date of HIV diagnosis. To account for any sociodemographic differences, we used dichotomous variables to classify education into “any postsecondary” vs “high school or less” based on the last reported education level and whether urban‐dwelling or not (rural, out‐of‐province, or unknown) based on residential postal codes.

### 2.4 Statistical analysis

#### 2.4.1 Ever testing

*Descriptive analyses*: We defined “ever testers” as those with at least one HCV test (serological or RNA) from all dates available and used descriptive statistics to characterize participants overall in the analytic sample (n = 4586 individuals) or by specific HCV exposure groups, defined by history of IDU and possible sexual transmission.

*Testing rate in HIV care by DAA era*: We calculated the annualized rate of having ever had an HCV test (serological or RNA), that is, the cumulative incidence of having ever tested for HCV in each year. To do so, we restricted analytic time to years under OCS follow‐up. We excluded participants who had an HCV test prior to baseline (1563 removed) or who had missing HCV test dates (six removed) for an analytic sample of 3017 individuals. Follow‐up ended at the date of the first HCV test or was censored at the last viral load test, last OCS visit, last date of OCS site data collection, or December 31, 2015, whichever was earliest. To test the effect of DAA approval on time to first HCV test under HIV care, we used Cox regression with robust standard errors, where DAA era was included as a time‐varying covariate. Using the Stata command ‐estat phtest‐, we tested the proportional hazards assumption based on Schoenfeld residuals. To address left truncation, we conducted a sensitivity analysis incorporating delayed entry (year of first HIV viral load test) and year of HIV diagnosis as the origin in a subsample including only those who tested for HCV prior to baseline and without restricting to OCS follow‐up (n = 5568).

#### 2.4.2 Annual serological testing for HCV

Among the analytic sample of 4586 individuals (39 337 person‐years), we calculated annual proportions of serological testing as the number of people with at least 1 HCV serological test in the calendar year that they also had a viral load test (“annual testers”) in the overall population as well as by HCV exposure groups, defined by history of any IDU. Follow‐up ended at the earlier date of December 31, 2015, last viral load test, last OCS visit, last date of OCS site data collection or, for those who eventually tested positive for HCV, the date of their first HCV‐positive test (serologic, RNA, or genotyping test). We identified correlates of annual testing using modified Poisson regression in a generalized estimating equations ^10^ framework with robust standard errors to account for repeated observations per person. Covariates were selected a priori and included in the final model if statistically significant (*P* < 0.05). Recent IDU, which was available only for a subset of participants who completed annual questionnaires after 2008, was used in a subanalysis.

#### 2.4.3 Number of serological tests per year

We used descriptive statistics and the nonparametric Wilcoxon signed rank tests for paired data to quantify and test for differences in number of HCV antibody tests per year by DAA era and transmission risk due to IDU or sexual contact. Follow‐up time was defined as above for annual serological testing.

#### 2.4.4 HCV diagnoses

Diagnosis was based on either laboratory tests (confirmed antibody test or positive RNA or genotype test) or medical records. Among participants whose HCV status was unknown or HCV‐negative at baseline, we calculated (1) the cumulative incidence of an HCV diagnosis and (2) the annual proportion diagnosed with HCV among all participants who were under observation and had a viral load test that year, whether or not they had an HCV test that year. Follow‐up ended at the HCV diagnosis date or was censored at the last viral load test, last OCS visit, last date of OCS site data collection, or December 31, 2015, whichever was earlier.

## 3 RESULTS

A total of 4586 participants were followed for a median of 9 years (interquartile range 4‐12 years; total 39 337 person‐years). The majority were male (84%), white (63%), living in urban settings (88%), and classified as MSM (64%) (Table 1). At baseline, 7.8% had a history of IDU; at follow‐up, 2.8% reported ongoing IDU, such that 11% had ever injected drugs by the end of follow‐up. At baseline, the median CD4 count was >350 cells and 72% were taking antiretroviral therapy (ART). By the end of follow‐up, 95% had initiated ART.

**TABLE 1:** Baseline^a^ characteristics of included participants from the Ontario HIV Treatment Network Cohort Study (OCS), n = 4586

	Study sample, *N* = 4586^b^
Sex	
Female	724 (15.8)
Male	3859 (84.2)
Age, years	40 (34‐47)
Any injection drug use^c^	359 (7.8)
HIV exposure category	
MSM	2930 (63.9)
MSM‐PWID	185 (4.0)
PWID	174 (3.8)
Heterosexual	559 (12.2)
Other^d^	738 (16.0)
Ethnicity/race	
White	2891 (63.0)
Black	722 (15.7)
Aboriginal/Indigenous	332 (7.2)
Other/Unknown	641 (14.0)
Region of residence	
Urban	4039 (88.1)
Rural/out of province/unknown	547 (11.9)
Year of HIV diagnosis	1998 (1992‐2005)
CD4 Cell Count/mm^3^	390 (235‐564)
Undetectable VL (≤50 copies)	1271 (27.7)
Initiated or on ART	3278 (71.5)

Abbreviations: MSM, men who have sex with men; MSM‐PWID, men who have sex with men and who inject drugs; PWID, people who inject drugs; VL, viral load; ART, antiretroviral therapy.

*Note*: Presented as n (%) or Median (Interquartile Range).

^a^ Baseline = later of January 1, 2000 or first viral load test or first OCS visit

^b^ Size of study sample for other outcomes differed. See Table S1 and main text for details.

^c^ Self‐reported or listed as HIV exposure category at baseline. By end of follow‐up, 486 (10.6%) individuals had ever reported injection drug use.

^d^ “Other” includes: clotting factor (0.8%), transfusion (1.1%), HIV‐endemic country (10.5%), mother‐to‐child transmission (0.3%), occupational (0.02%), nonidentified risk (3.4%).

### 3.1 Ever testing for HCV

At baseline, 34.1% (1563/4586) had a record of having had an HCV test. By the end of follow‐up, most (92.2%, 4227/4586) had tested at least once (“ever testers”); of the 3839 with complete first HCV test date records, 96.9% (3723/3839) had an antibody test only, 1.1% (41/3839) had an RNA test only, and 2.0% (75/3839) had both. The median time from HIV diagnosis and the first HCV test was 2 years (IQR 0‐9), with many (32.4%) testing for HCV in the same year of their HIV diagnosis. The proportion ever tested was highest among those with any report of IDU (97.1%, 472/486) and lowest among non‐MSM males with no history of IDU (90.2%, 514/570).

Of the 359 individuals who had no record of HCV testing, 4% reported any IDU and almost a quarter (22%) remained under follow‐up. On average at baseline, they were older than ever testers (43 vs 40) and had been living with HIV longer (7.5 vs 5.3 years).

### 3.2 HCV testing while in HIV care

Among the 2593 participants whose first HCV test occurred while under OCS follow‐up, the annualized rate of having ever had an HCV test was 70% higher after DAA approval (2011‐2015) compared to the pre‐DAA era (2000‐2010) (hazard rate ratio 1.70, 95% CI 1.59, 1.81). Proportional hazards assumptions held (*P* > 0.05). Results from the sensitivity analysis to address left truncation were similar (rate ratio = 1.16, 95% CI 1.06, 1.27). Prior to DAA approval, there were 28.5 HCV tests per 100 person‐years (95% CI 27.4, 29.8). After DAA approval, there were 46.6 HCV tests per 100 person‐years (95% CI 42.2, 51.3). After DAA approval, the mean time from HIV diagnosis to the first HCV test was also shorter compared to the pre‐DAA era (4.2 years vs 5.9 years, respectively), with 67.7% testing within 1 year of HIV diagnosis in the DAA era.

### 3.3 Annual HCV serological testing

**FIGURE 1:**
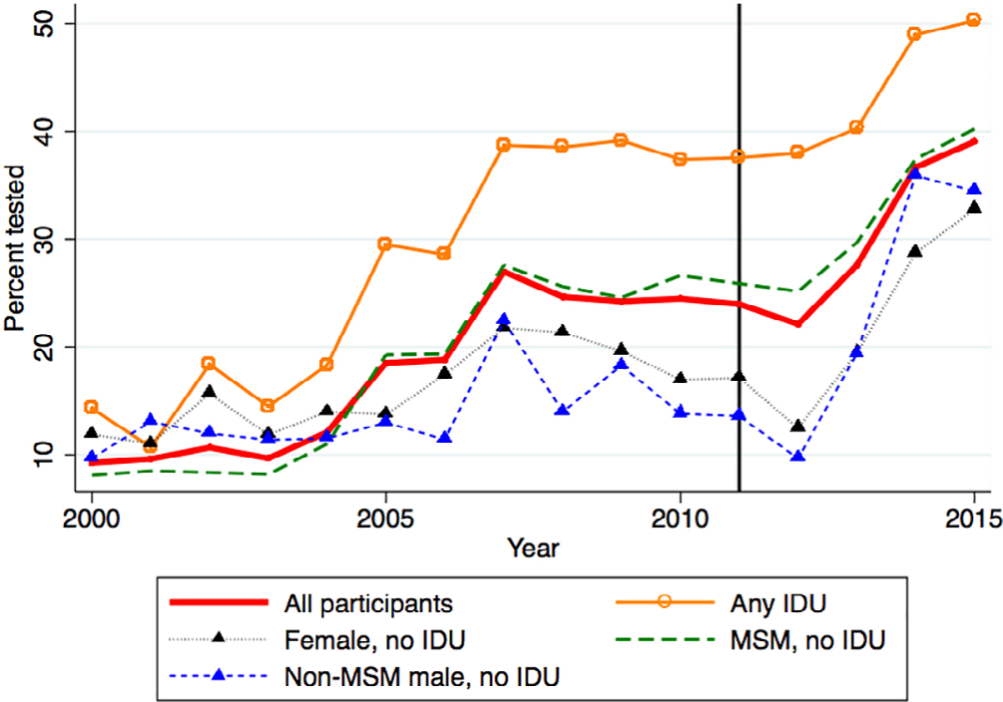
Annual proportion tested for Hepatitis C virus (HCV) antibodies in the OHTN Cohort Study by calendar year and HCV exposure group, 2000 to 2015. *Note*. Reference line: year 2011 when direct acting antivirals (DAAs) approved by Health Canada. Follow‐up ended at the earlier date of December 31, 2015, last viral load test, last OCS visit, last date of OCS site data collection or HCV diagnosis or first HCV‐positive test (either HCV‐antibody or RNA positive or record of any HCV genotype test). MSM, men who have sex with men; IDU, injection drug use; HCV, Hepatitis C virus; DAA, direct‐acting antivirals; OHTN, Ontario HIV Treatment Network

The annual proportion tested rose from 9.2% (95% CI 8.0%, 10.7%) in 2000 to a high of 39.1% (95% CI 37.1%, 41.1%) in 2015 (linear test of trend, *P* < 0.0001) (Figure 1). Testing increased by 18% per year post‐DAA, compared to 2% annual increases from 2000 to 2010 (Table 2). Results were similar when time was modeled using linear splines with knots at 2006 and 2011, cubic restricted spline, or a quadratic term (data not shown). In almost every calendar year, testing was most common among those with any history of IDU. The highest proportion was observed in 2015 among those who reported recent IDU (57.7%, 95% CI 36.9%‐76.6%).

**TABLE 2:** Selected correlates of annual testing for Hepatitis C virus (HCV) antibodies from included HIV‐positive participants in the OHTN Cohort Study, 2000 to 2015: results from generalized estimating equations^10^

	Unadjusted proportion ratio (95% CI)	Adjusted proportion ratio (95% CI)
Each additional calendar year^a^		
Pre‐DAA (2000‐2011)	1.00 (1.00, 1.01)	1.02 (1.01, 1.02)
Post‐DAA (2012‐2015)	1.16 (1.14, 1.18)	1.18 (1.16, 1.20)
Any injection drug use		
No	Referent	Referent
Yes	1.68 (1.55, 1.82)	1.42 (1.32, 1.53)
MSM		
Non‐MSM	Referent	Referent
MSM	1.25 (1.18, 1.33)	1.22 (1.13, 1.33)
Ever positive syphilis test		
No	Referent	Referent
Yes	1.31 (1.23, 1.39)	1.16 (1.09, 1.23)
Age, by decade	0.89 (0.87, 0.91)	0.90 (0.88, 0.93)
Sex		
Female	Referent	Referent
Male	1.19 (1.10, 1.28)	1.00 (0.91, 1.10)
Ethnicity/race		
White	Referent	Referent
Black	0.96 (0.89, 1.04)	0.96 (0.89, 1.04)
Aboriginal/Indigenous	1.13 (1.02, 1.25)	1.04 (0.94, 1.14)
Other/Unknown	1.10 (1.02, 1.19)	1.03 (0.96, 1.11)
Region		
Rural/out of province/unknown	Referent	Referent
Urban	1.28 (1.16, 1.42)	1.05 (0.95, 1.15)
Education		
High school or less	Referent	Referent
Post secondary	1.13 (1.06, 1.20)	1.07 (1.00, 1.13)
Duration of HIV, by decade	0.86 (0.82, 0.89)	0.81 (0.77, 0.84)

Abbreviations: DAA, direct‐acting antivirals; CI, confidence interval; MSM, men who have sex with men; OHTN, Ontario HIV Treatment Network.

^a^ Calendar time was modeled as a linear spline with a knot at 2011. Increase per calendar year was 16% higher in the post‐DAA era compared to pre‐DAA approval. Results similar when calendar time modeled differently (quadratic term or cubic spline).

Among those with no IDU history, on average, non‐MSM males and females were equally likely to test (16.5% and 18.4% per year, respectively) compared to MSM. Testing proportions did not differ statistically by sex or ethnicity after taking sexual or IDU risk factors into account (Table 2). Annual testing was more common among MSM or those who were ever diagnosed with syphilis or who reported any or recent IDU (adjusted proportion ratio [95% CI] in the subanalysis, 1.41 [1.22, 1.63]). Annual HCV testing was more common among urban‐dwellers and those with more education, but less common among older participants and those who had been living with HIV for a longer period of time. Results did not vary when study sites were included as categorical variables (results not shown).

### 3.4 Frequency of HCV serological testing

Among the 4227 individuals who had ever tested for HCV (serological or RNA), 3598 individuals had at least 1 antibody test over follow‐up. From 2000 to 2015, there were 11 077 HCV antibody tests among 3598 participants, for an average of 1.27 serological tests/person‐year; this was 4.8% higher (*P* < 0.05) in the DAA era compared to the pre‐DAA era (1.30 tests per year vs 1.24 tests per year, respectively). Among those at highest risk, we also observed increases in the mean number of annual serological tests following DAA approval. Among those with any history of IDU, the mean increased from 1.36 tests per year in 2000‐2010 to 1.49 tests per year in 2011‐2015 (9.6% rise), while among MSM with no history of IDU who had ever tested positive for syphilis, the average increased from 1.35 to 1.42 tests per year (5.2% increase). For groups considered “low‐risk,” such as non‐MSM males and females with no history of IDU, the mean number of annual serological tests changed slightly or not at all after DAA approval (0% and 4.1% increase, respectively).

### 3.5 HCV diagnosis

**FIGURE 2:**
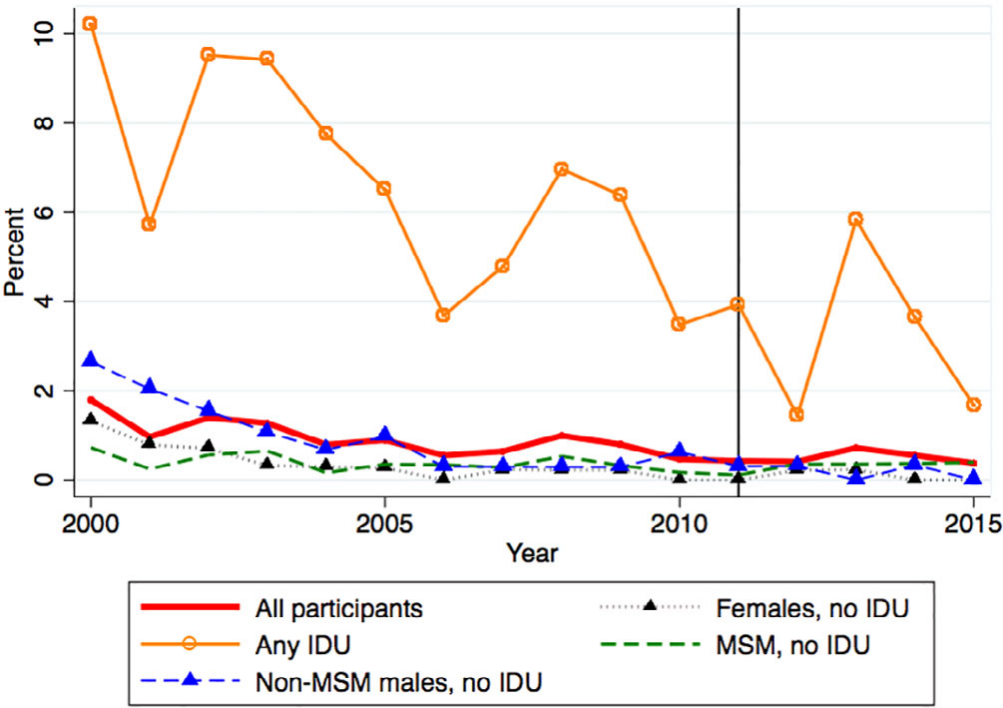
Annual proportion diagnosed^a^ with Hepatitis C virus (HCV) among included participants in the OHTN Cohort Study by calendar year and HCV exposure group, 2000 to 2015. *Note*. Reference line: year 2011 when direct acting antivirals (DAAs) approved by Health Canada. ^a^ Diagnosis based on either laboratory tests (confirmed antibody test or positive RNA or genotype test) or medical records. Denominator includes all participants who were enrolled in the OCS and had a viral load test that year, whether or not they had an HCV test that year. Follow‐up ended at the earlier date of December 31, 2015, last viral load test, last OCS visit, last date of OCS site data collection or HCV diagnosis or first HCV‐positive test (either HCV‐antibody or RNA positive or record of any HCV genotype test). MSM, men who have sex with men; IDU: injection drug use; HCV, Hepatitis C virus; DAA, direct‐acting antivirals; OHTN, Ontario HIV Treatment Network

While under follow‐up, 6.7% (305/4586) were diagnosed with HCV; this represented 8.5% (305/3598) of participants who had at least one HCV serological test over follow‐up. Among the 255 males diagnosed with HCV, 51.8% (132/255) reported a history of IDU; of those with no history of IDU, 74.0% (91/123) reported having sex with men but the remainder (26.0%, 32/123) did not. Among the 50 females diagnosed with HCV, 70.0% (35/50) reported a history of IDU but the remainder (30.0%, 15/50) did not. Unlike the annual proportion tested, the annual proportion diagnosed with HCV declined sharply from 1.8% (1.2%, 2.5%) in 2000 to 0.5% (0.2%, 0.8%) in 2010 and then more slowly to 0.4% (0.2%, 0.7%) by 2015 (Figure S1). In those with any IDU, the diagnosis rate dropped steeply and then more slowly; in the other subgroups, the HCV diagnosis rate stabilized after DAAs were approved in 2011 (Figure 2).

## 4 DISCUSSION

Among people engaged in HIV clinical care in Ontario, Canada, most had been tested for HCV at least once by 2015, with annual HCV testing proportions rising from 9.2% in 2000 to 39.1% by 2015. Following DAA approval in 2011, participants were both more likely to have ever tested and more likely to have tested within the past year. While the time between HIV diagnosis and the first HCV test was shorter after DAA approval, 32.3% tested in the DAA era did so more than 1 year after HIV diagnosis, presenting a significant gap as HCV acquisition can occur quickly (median time 1.25 years or 15 months) among those living with HIV.^11^ Reflecting earlier risk‐based testing guidelines in Canada and consistent with our hypotheses, annual testing was most common after DAA approval and in those at highest risk of acquiring or transmitting HCV either sexually or via IDU.

Since 2013, North American guidelines^12^ recommend annual HCV testing of HIV‐positive MSM and people actively injecting drugs. While these subgroups exhibited the highest improvements in testing over time in our study, only one‐third to half of those groups had tested annually by 2015. There are public health benefits if higher testing coverage among key subgroups leads to greater HCV treatment uptake and cure. Higher HCV prevalence (41.2% among those with any IDU in our study population) and denser drug‐using networks can lead to faster HCV transmission, re‐infection and persistence. A mathematical modeling study by Scott et al^13^ predicted that in medium‐prevalence settings (50% HCV sero‐prevalence among people who inject drugs, PWID), testing frequency and coverage had the greatest impact in reaching the WHO elimination target of 80% lower HCV incidence by 2030. The authors report that annual testing may be sufficient for elimination but only if coverage is 80% or higher. Otherwise, 6‐monthly testing coupled with higher retention in care (>60% retention of antibody‐positive patients) would be required. In our study population, no subgroup had such high levels of annual testing coverage, though the subset of individuals reporting recent IDU came close in 2015 (57.7%, 95% CI 36.9%‐76.6%). Only 14.6% of participants reporting any IDU had more than 1 test per year.

Targeted interventions to boost testing and care engagement may therefore be an effective tool to reach elimination goals, focusing on those with a history of IDU and sexual risk factors, with care taken to ensure equity in access. For example, we observed that annual testing was less common among older individuals in rural areas. Testing gaps in this population may have greater consequences at an individual, rather than public health, level; undiagnosed HCV in this group may lead to missed treatment opportunities and worse liver disease, for example, rather than engagement in ongoing transmission networks.

The drop in HCV diagnosis between 2000 and 2010 likely reflected changes in HCV treatment guidelines and uptake (pegylated interferon‐ribavirin was approved for HIV‐HCV co‐infected patients in 2003‐2004^14^) and is consistent with declining or stabilizing HCV incidence in the general population and subpopulations in Canada. In the Ontario general population,^15^ there was evidence of steeper declines of reported HCV cases between 2005 and 2009 with possible stabilization between 2010 and 2014.^16^ In specific subpopulations in the OCS, such as HIV‐positive MSM with no history of IDU, there was also no evidence for a temporal trend in HCV incidence between 2000 and 2010.^10^ Among PWID in British Columbia,^17^, ^18^ HCV incidence has been declining since 2000 due to changes in harm‐reduction and drug‐using practices.

There were limitations to our study. We may have under‐ascertained serologic HCV testing as this may have been carried out at private laboratories and would not be on record at the provincial public health laboratory (in the OCS, 35.6% of all HCV antibody tests and 100% of confirmatory tests was conducted by the PHOL). Such under‐ascertainment would produce underestimates of having ever tested and annual testing rates but would not vary by subgroups of interest (ie, nondifferential). Nevertheless, the public health laboratory conducts all confirmatory HCV testing, meaning that our approach should have captured all positive HCV antibody tests. Although the OCS is broadly representative of people in HIV care in Ontario, younger individuals and those who have recently acquired HIV are underrepresented ^19^ or those not in HIV care. Our results may be generalizable to other high‐income settings with universal health care.

In conclusion, our findings indicate that over time, annual HCV testing increased among individuals engaged in HIV care in Ontario, especially after DAA approval in 2011. While annual testing was higher in those with sexual or behavioral risk factors such as IDU, only about one‐third to half of HIV‐positive MSM and those who inject drugs had an HCV test in 2015. This is well below North American guidelines that recommend annual retesting of HIV‐positive MSM and active injection drug users and below testing coverage and frequencies needed to reach WHO elimination goals by 2030. For PWID, the subgroup most affected by HCV, targeted interventions to boost testing and care engagement for those who report recent IDU may be required to meet WHO elimination targets by 2030.

## CONFLICT OF INTEREST

None to declare.

## AUTHOR CONTRIBUTIONS

Conceptualization: Nasheed Moqueet, Ann Burchell.

Formal Analysis: Nasheed Moqueet, Sandra L. Gardner.

Funding Acquisition: Nasheed Moqueet, Ann Burchell.

Writing ‐Original Draft Preparation: Nasheed Moqueet with feedback from all authors.

Writing ‐ Review & Editing: All authors.

All authors have read and approved the final version of the manuscript.

Dr. Ann Burchell had full access to all of the data in this study and takes complete responsibility for the integrity of the data and the accuracy of the data analysis.

## TRANSPARENCY STATEMENT

Dr. Nasheed Moqueet and Ann Burchell (lead and senior author, respectively) affirm that this manuscript is an honest, accurate, and transparent account of the study being reported; that no important aspects of the study have been omitted; and that any discrepancies from the study as planned have been explained.

## Supporting information

**Figure S1.** Annual proportion with Hepatitis C virus (HCV) serological test, positive HCV testa, or HCV diagnosisb among included participants in the OHTN Cohort Study by calendar year, 2000 to 2015

**Table S1**. Definitions of outcomes and eligible study/analytic samples of included participants from the Ontario HIV Treatment Network Cohort Study (OCS)

## Data Availability

The data that support the findings of this study are not publicly available to protect the privacy of the participants but are available from the OHTN Cohort Study upon reasonable request and approval of the Governance Committee.
