# Supplementary material for: Hepatitis C virus testing in a clinical HIV cohort in Ontario, Canada, 2000 to 2015
Source: Health Sci Rep. 2021 Sep 18;4(3):e358. doi: 10.1002/hsr2.358 (PMC8449285; doi:10.1002/hsr2.358)
Supplement: Supplementary file 1 — Figure S1. Annual proportion with Hepatitis C virus (HCV) serological test, positive HCV testa, or HCV diagnosisb among included participants in the OHTN Cohort Study by calendar year, 2000 to 2015 [file HSR2-4-e358-s001.pdf]

**Supplementary Figure 1.** Annual proportion with Hepatitis C virus (HCV) serological test, positive HCV test[a], or HCV diagnosis[b] among included participants in the OHTN Cohort Study by calendar year, 2000-2015

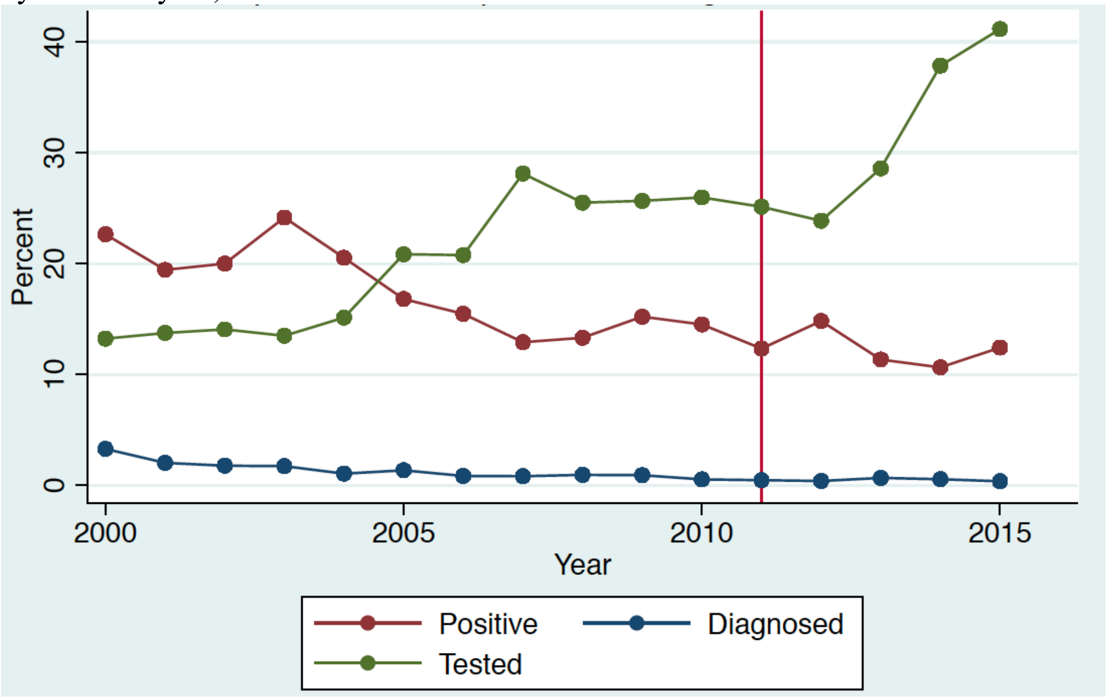


a. Numerator consists of positive antibody test or record of positive RNA or genotype test. Denominator includes those in the OCS who tested for HCV that calendar year. Positivity proportions, while allowing comparability to surveillance estimates as a proxy for incidence, are affected by changes in health seeking behaviors, service utilization and data quality and linkage.

b. Diagnosis based on either laboratory tests (confirmed antibody test or positive RNA or genotype test) or medical records. Denominator includes all participants who were enrolled in the OCS and had a viral load test that year, whether or not they had an HCV test that year.

Reference line: year 2011 when direct acting antivirals (DAAs) approved by Health Canada
