# Supplementary material for: Hepatitis C virus testing in a clinical HIV cohort in Ontario, Canada, 2000 to 2015
Source: Health Sci Rep. 2021 Sep 18;4(3):e358. doi: 10.1002/hsr2.358 (PMC8449285; doi:10.1002/hsr2.358)
Supplement: Supplementary file 2 — Table S1. Definitions of outcomes and eligible study/analytic samples of included participants from the Ontario HIV Treatment Network Cohort Study (OCS) [file HSR2-4-e358-s002.docx]

**Supplementary Table 1.** Definitions of outcomes and eligible study/analytic samples of included participants from the Ontario HIV Treatment Network Cohort Study (OCS)

|  | | **Definition** | | **Size of analytic sample** | |
| --- | --- | --- | --- | --- | --- |
| **From main text** | **Analysis from main text** | **Outcome** | **Eligible analytic person-time** | **Number of unique individuals** | **Person-years** |
| **‘Ever tested’** | *Descriptive* | Any HCV test (serological or RNA) at any time | ALL records from any time | n=4,586 | 39,711 |
|  | *Rate while in HIV care* | First any HCV test (serological or RNA) under OCS follow-up | Over study follow-up^a^ | n=3,017 | 8,525 |
|  | *Number of serological tests per year* | Only HCV serological tests per year under OCS follow-up | Among ever testers over study follow-up^a^ | n=3,598 | 8,750 |
| **‘Annual serological testing’** | *Proportions and proportion ratios* | At least 1 HCV serological test in the calendar year that they also had a viral load test | Over study follow-up^a^ | n=4,586 | 39,337 |

*^a. Study follow-up= years from baseline (later of Jan 1, 2000 or first viral load test or first OCS visit) to outcome or end of follow-up (earlier date of December 31, 2015, last viral load test, last OCS visit, or last date of OCS site data collection).^*
